# Signal Amplification in Highly Ordered Networks Is Driven by Geometry

**DOI:** 10.3390/cells11020272

**Published:** 2022-01-13

**Authors:** Éva S. Vanamee, Gábor Lippner, Denise L. Faustman

**Affiliations:** 1 Immunobiology Department, Massachusetts General Hospital, Harvard Medical School, Boston, MA 02129, USA; 2 Department of Mathematics, Northeastern University, Boston, MA 02115, USA; glippner@gmail.com

**Keywords:** TNF superfamily signaling, hexagonal clustering, Euler polyhedron formula

## Abstract

Here, we hypothesize that, in biological systems such as cell surface receptors that relay external signals, clustering leads to substantial improvements in signaling efficiency. Representing cooperative signaling networks as planar graphs and applying Euler’s polyhedron formula, we can show that clustering may result in an up to a 200% boost in signaling amplitude dictated solely by the size and geometry of the network. This is a fundamental relationship that applies to all clustered systems regardless of its components. Nature has figured out a way to maximize the signaling amplitude in receptors that relay weak external signals. In addition, in cell-to-cell interactions, clustering both receptors and ligands may result in maximum efficiency and synchronization. The importance of clustering geometry in signaling efficiency goes beyond biological systems and can inform the design of amplifiers in nonbiological systems.

## 1. Introduction

Membrane receptors allow cells to respond to external stimuli, and earlier models have predicted the homogeneous arrangement of receptors on the cell surface for optimal ligand binding and activation. However, this is in contrast to a growing body of experimental evidence suggesting that signaling efficiency can be greatly improved by the clustering of receptors [1,2]. Furthermore, recent super-resolution optical microscopy data have shown that many receptors preferentially organize in nanoclusters on the cell surface even in the absence of activation or ligand binding [3,4]. Nanoclustering of receptors may, therefore, be a common feature of the plasma membrane. The factors that regulate and stabilize nanoclusters are of considerable interest. In lipid-linked and transmembrane protein clusters, receptors are primarily stabilized by *cis* interactions between the ectodomains and cytosolic domains or via lipid anchors. Examples include members of the TNF receptor superfamily (TNFRSF) that cluster via their pre-ligand assembly domain (PLAD) [5,6,7]. There is mounting evidence that TNF receptors and their downstream signaling partners organize into hexagonal lattices [7,8,9,10,11,12]. Other examples of hexagonal clustering include chemo- or phototaxis receptors [2,13,14]. These represent examples of receptors that are able to self-assemble on the cell surface on the basis of their inherent symmetry, although this does not rule out the participation of other proteins in the process. Interestingly, pentameric gamma-aminobutyric acid A receptor (GABA(A)R) and glycine receptor (GlyR), which are unable to form 2D lattices by themselves, can also organize into hexagonal clusters on the cell surface by binding to a hexagonal scaffold formed under the cell membrane by gephyrin [15,16,17]. Further scaffolding examples include the spectrin–ankyrin hexagonal–triangular clusters [18] that form a network under the cell membrane of eukaryotic cells and control the surface display and localization of proteins in the plasma membrane [19]. Hexagonal networks are special for several reasons. Regular hexagons are the highest-order polygons that can be tiled or tessellated in a regular pattern by themselves, where each hexagon is surrounded by six other hexagons, repeated indefinitely in any direction with no gaps in between. Hexagonal tiling uses the smallest perimeter to enclose a particular area in space [20]. Therefore, the use of a hexagonal network is the most economical way to build a network.

On the basis of the growing experimental evidence for clustering, more recent theoretical models have proposed that the optimal solution to the biological information-processing problem has to balance two opposing objectives: on the one hand, to reduce noise as the receptors need to concentrate locally; on the other hand, to provide broader coverage as the receptors need to be spread out [21]. To our knowledge, to date, no one has explored what role the clustering geometry might play in signaling. Here, we hypothesize that clustering may result in signal amplification that depends on cluster size and geometry and illustrate how ordered nanoclustering can provide an optimal solution to the biological information-processing problem

## 2. Methods

Hexagonal networks play an important role in many applications, and methods to calculate the number of edges and vertices were described earlier [22]. The equations to calculate the ***e***/***n*** ratio for the specific examples shown in Figure 2 are detailed in Appendix A. 

## 3. Results and Discussion

**Figure 1:**
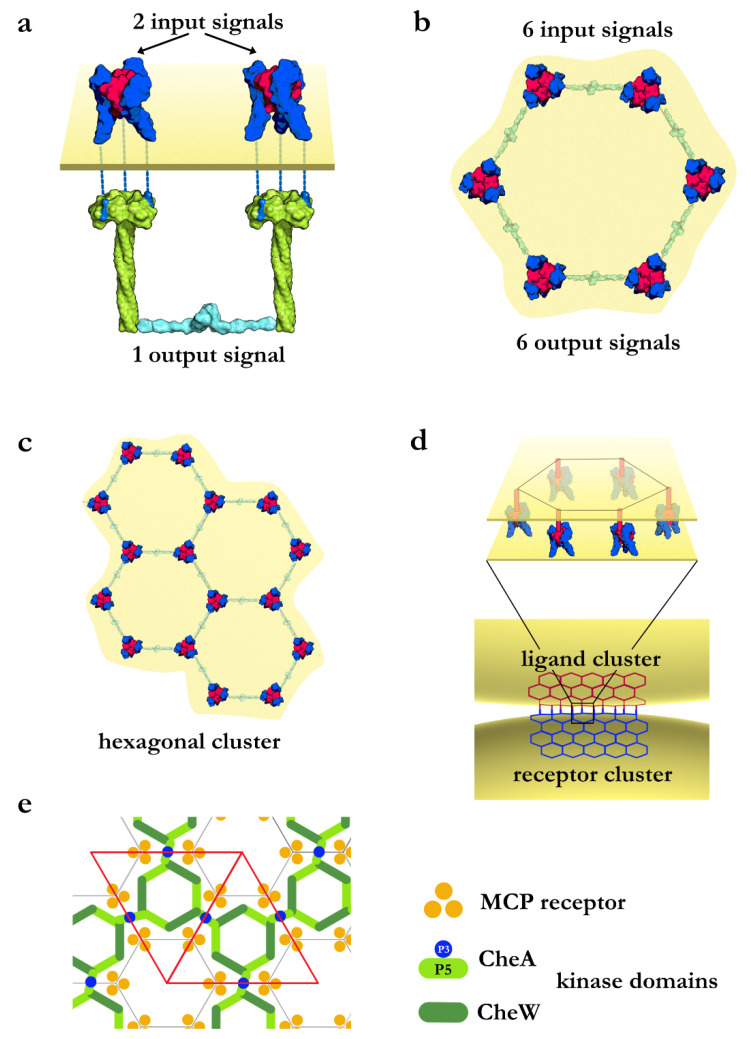
Illustration of cooperative biological signaling systems. (**a**) The TNF/TNFR2/TRAF signaling complex. The input signal is represented by TNF ligand (magenta) binding to TNFR2 (blue). Receptor activation initiates TRAF2 (green) recruitment and dimerization via the TRAF2 N-terminal RING domains (cyan) that represents the output signal. Connecting two signaling units introduces cooperativity, but the overall output signal is only half of the original input signals. (**b**) When six signaling units are connected in a hexagon (top view), the strength of the input signal can be maintained. (**c**) The hexagonal unit can further cluster into an ordered hexagonal signaling network. (**d**) In cell-to-cell interactions, clustered ligands attached to the membrane allow the simultaneous activation of clustered receptors with the same geometry, resulting in maximum signaling efficiency. (**e**) Chemotaxis MCP receptor baseplate. The MCP receptor trimers are connected by the CheA kinase dimers (blue) to introduce cooperativity. The triangular unit cell is shown in red.

We first consider receptors on a cell surface that are arranged randomly, independent of each other. Receptors can either be inactive (0 state) or activated by their ligands (1 state). The signal in such a random disconnected network travels vertically without synchronization between the receptors, and the overall amplitude of a signal (i.e., the sum of all the ones and zeros) generated by such a system is low, as, at any given time, only a small percentage of receptors will be active. In addition, because of the asynchronous nature of the signal, it will be distributed broadly over time. Next, we consider two receptor complexes (A and B) physically connected to create a new output signal, wherein the connection (AB) is only made when both receptors A and B are activated by their input signal, the ligand (AB = 1 if A and B = 1, and AB = 0 if A or B = 0). In essence, this is a logical AND gate and requires the synchronization between two input signals. An example is downstream tumor necrosis factor receptor-associated factor (TRAF) dimerization upon TNF receptor 2 (TNFR2) activation of two adjacent receptor trimers (Figure 1a). The connection creates a single output from two input signals. AB can only reach a maximum of 50% of the signal strength of the original signals A and B since there is a single connection between the two input signals. However, if six signaling units were connected in a closed loop, the signal strength of the input signal can be maintained (Figure 1b). That is because closed loops have an equal number of edges (e) and vertices or nodes (n). We can achieve further gains in efficiency by simultaneously activating the receptors by their ligands, which eliminates the stochasticity. In the case of TNF receptors, simultaneous activation requires saturating the receptor sites with soluble ligands [23] or using localized ligands arranged in the same geometry as the receptors (Figure 1d). Most TNF ligands are type II transmembrane proteins expressed in a membrane-bound form and can be cleaved to produce soluble ligands. The membrane-bound ligands are known to signal more effectively via their receptors than the soluble ligands [24]. This has been demonstrated experimentally in a model system of the apoptosis inducing receptor CD95 (Fas), another member of the TNF receptor superfamily, and its ligand CD178 (FasL). Using a DNA origami platform, the FasL ligands were immobilized and arranged in different geometries to test the effect of clustering on apoptosis efficiency in HeLa cells overexpressing the Fas receptor. Hexagonally arranged ligands signaled orders of magnitude better than soluble ligands [25]. Furthermore, ligands arranged in a hexagon with the right geometry doubled the rate of apoptosis compared to ligands arranged in pairs [25]. All of this indicates that membrane-bound ligands achieve more efficient signaling by having the same clustering geometry on the cell surface as the receptors. As we described earlier, members of the TNF receptor superfamily are further arranged in a hexagonal lattice on the cell surface [7,26]. 

Beyond building a stable network, clustering with a specific geometry has other important benefits, as illustrated below. 

Cooperative signaling networks can be represented as planar graphs, i.e., a G planar graph with ***n*** nodes (degrees: ***d_1_***, ***d_2_***, **d_3_**, …, ***d_n_***). The degree of a node represents the number of edges meeting at that node.

(1) $$e\;\mathrm{edges}\;(e=\frac{1}{2}\sum_{j=1}^{n}d_{j}).$$

This graph has ***f*** faces (face sides: ***s_1_***, **s_2_**, ***s_3_***, …, ***s*_f_**). Each face contributes ***s_j_*** face sides or edges; however, because each edge is shared by two faces, the sum total has to be divided by 2.

(2) $$e=\frac{1}{2}\overset{f}{\underset{j=1}{\sum }}s_{j}.$$

(3) $$\mathrm{For}\;s_{j}\geq 3,\;e\geq \frac{3f}{2}.$$

Replacing ***f*** with Euler’s polyhedron formula [27],

(4) f=e−n+2,

yields

(5) e≤3n−6..

In a “simple” graph with no self-loops or multiple edges, for each planar graph with $s_{j}\geq 3.$ and n≫1,

(6) $$\frac{e}{\;n}\leq 3$$

For clustered signaling systems, illustrated by tiled polygons, the ***e***/***n*** ratio represents the ratio of the output signal over the input signal. According to Equation (6), the ***e***/***n*** ratio can never exceed 3. In practical terms, what this means is that the input signal in a clustered network is amplified, and a maximum of three times the original amplitude or a 200% gain in amplification can be achieved. The actual number depends on the geometry and size of the cluster.

**Figure 2:**
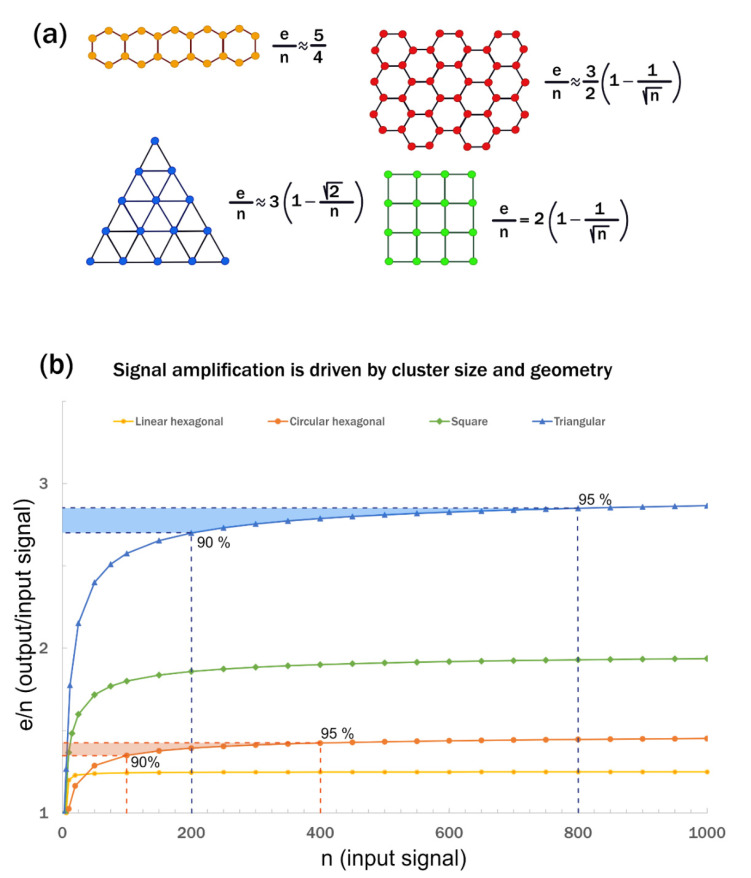
Illustration of Euler’s formula for 2D polygons. (**a**) The ******e***/***n****** ratio is calculated for several examples of regular tiled polygons. (**b**) The ******e***/***n****** ratio plotted against n for the tiled polygonal systems shown in (**a**). It represents signal amplification and reaches its maximum level with tiled triangles but can never exceed 3. A 90% maximum signal amplification can be achieved with a cluster of ~100 signaling units in a hexagonal cluster depending on geometry, but the same level of amplification requires a cluster of ~200 signaling units in a triangular cluster. Calculations are provided in Appendix A.

As we described earlier, the clustering of TNF receptors on the membrane surface can be illustrated by tiled regular hexagons. The input signal is represented by the vertices or nodes, and the output signal is represented by the edges of the hexagon. Figure 2a illustrates two scenarios of hexagonal tiling. As the cluster size grows, the ******e***/***n****** ratio increases and reaches a plateau (Figure 2b). This implies that, in addition to the maximum amplification, there is also an optimum cluster size for amplification beyond which the input/output ratio does not increase considerably. For hexagonal clusters, 90% of the maximum signal amplification can be achieved in a cluster of 100 signaling units and 400 units to reach 95% signal amplification. It takes 200 and 800 signaling units to reach the 90% and 95% ***e***/***n*** levels, respectively, in a triangular cluster (Figure 2b). This is important because experimental data indicate that signaling proteins tend to form small nanoclusters on the cell surface around 300–500 nm in diameter [4,15,28]. Optimal cluster size may also depend on the size of the cell-to-cell interface. According to the numbers presented here, a cluster of 100–200 receptors is sufficient to reach 90% of the maximum level of amplification. The signal amplitude also depends on the geometry of the cluster. Tiling the hexagons in a more or less symmetrical fashion in each direction is the most efficient, leading to the highest ***e***/***n*** or input/output signal ratio compared to hexagons tiled in a linear fashion. This is because the ***e***/***n*** ratio is maximized when most hexagons are surrounded by other hexagons or, in other words, when the number of ***d_j_*** nodes with maximum connections is the highest.

In general, in the case of polygonal tiling in 2D, ***d_j_*** in Equation (1) is not constant; therefore, we can introduce ***d_ave_***, which can be calculated as

(7) $$d_{ave}=\frac{1}{n}\overset{n}{\underset{j=1}{\sum }}d_{j}.$$

Equation (1) can then be rewritten as

(8) $$e=\frac{n\;d_{ave}}{2},$$

and

(9) $$\frac{e}{n}=\frac{d_{ave}}{2}.$$

For nodes of polygons on the inside of the cluster, ***d = d_max_***, whereas nodes on the outside of the cluster have a ***d*** value smaller than ***d_max_***, leading to ***d_ave_ < d_max_***. As ***n*** increases, ***d_ave_*** converges on ***d_max_***. For tiled hexagons, a maximum of three edges can be joined together at each node leading to a ***d_max_*** of 3, translating to a maximum amplitude of 1.5 times the original signal in hexagonally clustered receptors. The ***e***/***n*** ratio is also inversely correlated with the degree of the polygon, and it is highest for tiled triangles with a ***d_max_*** of 6 (Figure 2b).

From our analysis, the hexagonal lattice of TNF receptors is optimized for signal transduction. It provides the most economical way to build a stable scaffold. Beyond simply building the most efficient network, however, clustering not only maintains the input signal but also boosts it by its specific geometry. The growing number of hexagonal biological systems indicates that this may be a common arrangement of signaling networks in general. In addition to TNF receptors and their downstream signaling partners, chemo- or phototaxis receptors also cluster into hexagonal core complexes, consisting of trimers of dimers that further assemble to form large hexagonal arrays [2,13,14]. Signal amplification has also been observed in these receptors, and it has been proposed that the amplification is the result of cooperativity in the clustered arrays [14,29,30]. Interestingly, in the case of *E. coli* methyl-accepting chemotaxis protein (MCP) receptors, the histidine kinase (CheA) elements responsible for cooperativity are arranged to create triangular repeats within the hexagonal lattice, potentially to maximize signal amplification (Figure 2e) [2]. We hypothesize that, in chemo- and photoreceptors that sense weak external signals, the geometry is optimized to achieve maximum amplification, which may be crucial to generate an appropriate cellular response. Mimicking natural receptor clustering, artificial two-dimensional scaffolds have now been developed that utilize hexagonal lattices to modulate cell responses [31].

## 4. Conclusions

In summary, signaling efficiency can be greatly improved in cooperative signaling networks by clustering receptors on the cell surface. We can simplify such cooperative networks as planar graphs, where the input signal is represented by the vertices or nodes, the output signal is represented by the edges, and the relationship between them is determined by Euler’s polyhedron formula. As the cluster size grows, the output signal is amplified with respect to the input signal, and the maximum level of amplification, represented in Equation (9), is determined by cluster size and geometry. Plotting the ***e***/***n*** ratio against the number of signaling units or cluster nodes illustrates that there is an optimal cluster size beyond which the relative increase in amplitude becomes negligible. Maximum signal amplification can be achieved in triangular clusters. The TNF receptor signalosome is optimized for maximum synchronicity by clustering both the receptors and ligands with the same geometry. This allows simultaneous activation of the receptors and results in a sharp digital-like output signal. In chemo- and photoreceptors that sense random diffuse signals the geometry seems to be optimized for maximum amplification. Despite its simplicity, Euler’s polyhedron formula is a deep theorem with numerous applications in geometry, topology, dynamical systems, and medicine [32]. We have now shown it to be essential to describe signal amplification in biological signaling networks. How broadly this relationship will apply remains to be seen. Signaling systems can deliver both activating and inhibitory signals, and our model can apply to both scenarios. Another important question is how the process of clustering and cluster size is regulated. It has been proposed that the dynamic modeling and remodeling of skeletal proteins such as actin regulates the spatiotemporal organization of cell surface molecules [33,34]. In addition, molecules may be actively transported to the cell surface by other proteins. An example is the display and localization of CD45 by the spectrin–ankyrin skeleton [35]. Cluster size maybe determined simply by the entropic cost of growing the cluster or by skeletal proteins that function as a “fence” to inhibit cluster growth beyond a certain size [3]. In addition, coregulatory proteins may aid or interfere with the clustering of other proteins and, therefore, modulate the amplitude of the output signal. For instance, members of the TNF receptor superfamily directly interact with members of the immunoglobulin superfamily (IgSF), which function as coregulatory proteins to modulate cluster formation [36]. As we discussed earlier, nanoclusters of 100–200 signaling units, depending on geometry, are sufficient to reach 90% of maximum signal amplification. Homogeneously distributing small nanoclusters on the cell surface instead of individual molecules may, therefore, provide an optimum solution to the information-processing problem posed earlier. We believe that many other signaling systems will be identified in the future where clustering with a specific geometry is necessary to improve signaling efficiency. Top candidates include proteins of the cytoskeleton that may also play a functional role in signal transduction [37,38]. The importance of these findings goes beyond biological systems and could influence the design of amplifiers in electronics and in light sensors, among other systems.

## Appendix A

In the following examples the ***e***/***n*** ratio is calculated for tiled regular polygons with different geometry:
**Example** **A1.** *Hexagonal tiling with k number of hexagons arranged in a line shown in Figure 2a:*

**e** **=** **6** **+** **(k** **−** **1)** **×** **5,** **n** **=** **6** **+** **(k** **−** **1)** **×** **4,**

*When* $n\gg 6,\;\frac{e}{n}\approx \frac{5}{4}.$
**Example** **A2.** *Hexagonal tiling where a and b are the number of horizontally and vertically tiled hexagons, respectively:*

$$e=\frac{1}{2}\left(6ab+4a-2+4b\right),\;n=\left(a+1\right)\cdot \left(2b+2\right)-2,\;\frac{e}{n}=\frac{3ab+2a+2b-2}{2ab+2b+2a}=\frac{3}{2}-\frac{a+b+2}{n}.$$

*If* a = 2b − 1, *then*

$$\frac{e}{n}\approx \frac{3}{2}-\frac{3}{2\sqrt{n}}=\frac{3}{2}\left(1-\frac{1}{\sqrt{n}}\right).$$

*When* $n\gg 1,\;\frac{e}{n}\approx \frac{3}{2}$.
**Example** **A3.** *Square tiling shown in Figure 2a where **k** is the number of square edges on each side:*

**e = 2k (k + 1),**

$$n={\left(k+1\right)}^{2}\Rightarrow k=\sqrt{n}-1,$$

$$\frac{e}{n}=\frac{2k\left(k+1\right)}{\left(k+1\right)\left(k+1\right)}=\frac{2\left(\sqrt{n}-1\right)}{\sqrt{n}}=2-\frac{2}{\sqrt{n}}.$$

*When* $n\gg 1,\;\frac{e}{n}\approx \;2$.
**Example** **A4.** *Triangle tiling illustrated in Figure 2a where **k** is the number of tiles on each side:*

$$e=\frac{1}{2}\left[3k+3\left(1+3+5+\cdots +2k+1\right)\right]=\;\;\frac{3}{2}\left[k^{2}+k\right]=\frac{3}{2}\;k\;\left(k+1\right),\;n=1+2+\cdots +k+1=\frac{\left(k+1\right)\cdot \left(k+2\right)}{2},\;\frac{e}{n}=\frac{3k}{k+2}\approx 3-\frac{6}{\sqrt{2n}}.$$

*When* $n\gg 1,\;\frac{e}{n}\approx 3$. *In each example, as n increases, the **e**/**n** ratio approaches the value of* $\frac{d_{max}}{2}$.

## References

[1] Bray D., Levin M.D., Morton-Firth C.J. (1998). Receptor clustering as a cellular mechanism to control sensitivity. Nature.

[2] Briegel A., Li X., Bilwes A.M., Hughes K.T., Jensen G.J., Crane B.R. (2012). Bacterial chemoreceptor arrays are hexagonally packed trimers of receptor dimers networked by rings of kinase and coupling proteins. Proc. Natl. Acad. Sci. USA.

[3] Garcia-Parajo M.F., Cambi A., Torreno-Pina J.A., Thompson N., Jacobson K. (2014). Nanoclustering as a dominant feature of plasma membrane organization. J. Cell Sci..

[4] Sherman E., Barr V., Samelson L.E. (2013). Super-resolution characterization of TCR-dependent signaling clusters. Immunol. Rev..

[5] Naismith J.H., Devine T.Q., Brandhuber B.J., Sprang S.R. (1995). Crystallographic evidence for dimerization of unliganded tumor necrosis factor receptor. J. Biol. Chem..

[6] Chan F.K., Chun H.J., Zheng L., Siegel R.M., Bui K.L., Lenardo M.J. (2000). A domain in TNF receptors that mediates ligand-independent receptor assembly and signaling. Science.

[7] Vanamee E.S., Faustman D.L. (2018). Structural principles of tumor necrosis factor superfamily signaling. Sci. Signal..

[8] Scott F.L., Stec B., Pop C., Dobaczewska M.K., Lee J.J., Monosov E., Robinson H., Salvesen G.S., Schwarzenbacher R., Riedl S.J. (2009). The Fas-FADD death domain complex structure unravels signalling by receptor clustering. Nature.

[9] Yin Q., Lin S.C., Lamothe B., Lu M., Lo Y.C., Hura G., Zheng L., Rich R.L., Campos A.D., Myszka D.G. (2009). E2 interaction and dimerization in the crystal structure of TRAF6. Nat. Struct. Mol. Biol..

[10] Napetschnig J., Wu H. (2013). Molecular basis of NF-kappaB signaling. Annu. Rev. Biophys..

[11] Graves J.D., Kordich J.J., Huang T.H., Piasecki J., Bush T.L., Sullivan T., Foltz I.N., Chang W., Douangpanya H., Dang T. (2014). Apo2L/TRAIL and the death receptor 5 agonist antibody AMG 655 cooperate to promote receptor clustering and antitumor activity. Cancer Cell.

[12] Zapata J.M., Perez-Chacon G., Carr-Baena P., Martinez-Forero I., Azpilikueta A., Otano I., Melero I. (2018). CD137 (4-1BB) Signalosome: Complexity Is a Matter of TRAFs. Front. Immunol..

[13] Yang W., Alvarado A., Glatter T., Ringgaard S., Briegel A. (2018). Baseplate variability of Vibrio cholerae chemoreceptor arrays. Proc. Natl. Acad. Sci. USA.

[14] Orekhov P., Bothe A., Steinhoff H.J., Shaitan K.V., Raunser S., Fotiadis D., Schlesinger R., Klare J.P., Engelhard M. (2017). Sensory Rhodopsin I and Sensory Rhodopsin II Form Trimers of Dimers in Complex with their Cognate Transducers. Photochem. Photobiol..

[15] Specht C.G., Izeddin I., Rodriguez P.C., El Beheiry M., Rostaing P., Darzacq X., Dahan M., Triller A. (2013). Quantitative nanoscopy of inhibitory synapses: Counting gephyrin molecules and receptor binding sites. Neuron.

[16] Sola M., Bavro V.N., Timmins J., Franz T., Ricard-Blum S., Schoehn G., Ruigrok R.W., Paarmann I., Saiyed T., O’Sullivan G.A. (2004). Structural basis of dynamic glycine receptor clustering by gephyrin. EMBO J..

[17] Hoffmann C., Milovanovic D. (2021). Gephyrin: A scaffold that builds a phase at the inhibitory postsynapses. Cell Res..

[18] Pan L., Yan R., Li W., Xu K. (2018). Super-Resolution Microscopy Reveals the Native Ultrastructure of the Erythrocyte Cytoskeleton. Cell Rep..

[19] Machnicka B., Czogalla A., Hryniewicz-Jankowska A., Boguslawska D.M., Grochowalska R., Heger E., Sikorski A.F. (2014). Spectrins: A structural platform for stabilization and activation of membrane channels, receptors and transporters. Biochim. Biophys. Acta.

[20] Hales T.C. (2001). The honeycomb conjecture. Discret. Comput. Geom..

[21] Iyengar G., Rao M. (2014). A cellular solution to an information-processing problem. Proc. Natl. Acad. Sci. USA.

[22] Prvan M., Ožegovic J., Mišura A.B. (2019). A Review of Embedding Hexagonal Cells in the Circular and Hexagonal Region of Interest. Int. J. Adv. Comput. Sci. Appl. (IJACSA).

[23] Tay S., Hughey J.J., Lee T.K., Lipniacki T., Quake S.R., Covert M.W. (2010). Single-cell NF-kappaB dynamics reveal digital activation and analogue information processing. Nature.

[24] Grell M., Douni E., Wajant H., Lohden M., Clauss M., Maxeiner B., Georgopoulos S., Lesslauer W., Kollias G., Pfizenmaier K. (1995). The transmembrane form of tumor necrosis factor is the prime activating ligand of the 80 kDa tumor necrosis factor receptor. Cell.

[25] Berger R.M.L., Weck J.M., Kempe S.M., Hill O., Liedl T., Radler J.O., Monzel C., Heuer-Jungemann A. (2021). Nanoscale FasL Organization on DNA Origami to Decipher Apoptosis Signal Activation in Cells. Small.

[26] Torrey H., Butterworth J., Mera T., Okubo Y., Wang L., Baum D., Defusco A., Plager S., Warden S., Huang D. (2017). Targeting TNFR2 with antagonistic antibodies inhibits proliferation of ovarian cancer cells and tumor-associated Tregs. Sci. Signal..

[27] Euler E. (1758). Elementa doctrine solidorum. Novi Comment. Acad. Sci. Petropolitanae.

[28] Siegel R.M., Muppidi J.R., Sarker M., Lobito A., Jen M., Martin D., Straus S.E., Lenardo M.J. (2004). SPOTS: Signaling protein oligomeric transduction structures are early mediators of death receptor-induced apoptosis at the plasma membrane. J. Cell Biol..

[29] Li M., Hazelbauer G.L. (2014). Selective allosteric coupling in core chemotaxis signaling complexes. Proc. Natl. Acad. Sci. USA.

[30] Sourjik V., Berg H.C. (2004). Functional interactions between receptors in bacterial chemotaxis. Nature.

[31] Ben-Sasson A.J., Watson J.L., Sheffler W., Johnson M.C., Bittleston A., Somasundaram L., Decarreau J., Jiao F., Chen J., Mela I. (2021). Design of biologically active binary protein 2D materials. Nature.

[32] Richeson D.S. (2008). Euler’s Gem: The Polyhedron Formula and the Birth of Topology.

[33] Gowrishankar K., Ghosh S., Saha S., Rumamol C., Mayor S., Rao M. (2012). Active remodeling of cortical actin regulates spatiotemporal organization of cell surface molecules. Cell.

[34] Saha S., Lee I.H., Polley A., Groves J.T., Rao M., Mayor S. (2015). Diffusion of GPI-anchored proteins is influenced by the activity of dynamic cortical actin. Mol. Biol. Cell.

[35] Pradhan D., Morrow J. (2002). The spectrin-ankyrin skeleton controls CD45 surface display and interleukin-2 production. Immunity.

[36] Vanamee E.S., Faustman D.L. (2020). On the TRAIL of Better Therapies: Understanding TNFRSF Structure-Function. Cells.

[37] Forgacs G., Yook S.H., Janmey P.A., Jeong H., Burd C.G. (2004). Role of the cytoskeleton in signaling networks. J. Cell Sci..

[38] Colin A., Bonnemay L., Gayrard C., Gautier J., Gueroui Z. (2016). Triggering signaling pathways using F-actin self-organization. Sci. Rep..

